# Experimental diagenesis reveals preservation of biosignatures in filamentous sulfur mats under hydrothermal conditions

**DOI:** 10.1038/s41598-025-25172-4

**Published:** 2025-10-31

**Authors:** Hrvoje Višić, Jan-Peter Duda, Stefan Fischer, Cristina Escudero, Fatih Sekerci, Andreas Kappler, Muammar Mansor

**Affiliations:** 1 Department of Geosciences, University of Tübingen, Tübingen, Germany; 2 Department of Geobiology, Geoscience Center, University of Göttingen, Göttingen, Germany; 3 Tübingen Structural Microscopy Core Facility, University of Tübingen, Tübingen, Germany; 4 Cluster of Excellence EXC 2124, Controlling Microbes to Fight Infection, University of Tübingen, Tübingen, Germany

## Abstract

Filamentous microbial biosignatures associated with iron sulfides are among the prime targets in early life studies, but their formation and preservation are insufficiently understood. Here, we experimentally evaluated the taphonomy of filamentous sulfur-oxidizing bacteria exposed to iron–sulfur–rich conditions and high temperatures (≤ 80 °C), mimicking burial diagenesis and/or hydrothermal alteration. The addition of ferrihydrite and sulfide at 22 °C resulted in a near-instantaneous formation of iron sulfides. Heating to 80 °C for 2–6 weeks resulted in the formation of polysulfides and magnetic Fe- and/or S-containing minerals, with low pyritization (~ 11%). Notably, Fe–S mineral formation was only loosely associated with the filaments. However, intracellular elemental sulfur released from the sulfur-oxidizing bacteria re-precipitated extracellularly, coating individual filaments, possibly promoting the formation of pyritic crusts during later diagenetic stages. Taken together, our study revealed that biosignatures in filamentous sulfur mats might be preserved in a variety of environments, including hydrothermal systems on and beyond the Earth.

**Supplementary Information:**

The online version contains supplementary material available at 10.1038/s41598-025-25172-4.

## Introduction

Biosignatures (i.e., objects, substances, and/or patterns whose origin specifically requires a biological agent^1^) are central for exploring the evolution of life on Earth and beyond. Morphological biosignatures indicative of microorganisms can be macroscopic (e.g., laminated structures formed by benthic microbial communities) or microscopic in scale (e.g., microfossils of filaments or individual cells). The interpretation of such biosignatures is commonly controversial, as exemplified by reports of putative microbial microfossils in > 3.4 billion years (Ga) old rocks^2–4^. First, microfossils might be confused with morphologically similar structures formed by abiotic processes, i.e. “biomorphs”^4–7^. Indeed, organic biomorphs formed under silica or sulfur (S⁰) rich conditions can be morphologically and chemically very similar to microfossils, sounding a note of caution that some putative microbial fossils in early Precambrian rocks are actually pseudofossils^5^. Second, morphological structures related to microorganisms may be destroyed by a range of processes occurring during or after deposition^8,9^. Hence, understanding the formation of geologically stable morphological biosignatures is a critical prerequisite to extracting information about life from the ancient rock record.

Morphological biosignatures associated with sulfide minerals such as pyrite (Py, FeS_2_) are one of the prime targets in early life studies. Many of these minerals can be preserved over geological time scales and, at the same time, potentially provide information about microbial metabolism involved in their formation and preservation (e.g., sulfate-reducers or S⁰-oxidizers). For instance, laminated microbialites consisting of metal sulfides such as Py and sphalerite (ZnS) from a hydrothermal environment of the 3.5 Ga Dresser Formation in Western Australia (WA)^10^ were interpreted to have formed through microbial processes^11–13^. Another example is framboidal-like Py preserved in a microbial mat facies from the 3.4 Ga Strelley Pool Formation in WA, potentially indicating microbial S⁰ and/or Fe cycling^14^. Notably, pyritic filaments (0.5–2.0 μm in diameter) in hydrothermal sulfides of the ~ 3.2 Ga Sulphur Springs Group in WA – perhaps the oldest black smoker deposit on Earth^15^ – were interpreted as microbial microfossils^16^. Furthermore, putative filamentous pyritic microfossils in the same diameter range (0.5–2.0 μm) were reported from the 1.4 Ga Gaobanhe hydrothermal sulfide deposit in China^17^ as well as from ~ 518 million years (Ma) old Cambrian and ~ 270 Ma old Permian non-hydrothermal deposits (Shuijingtuo Formation, China and Glass Mountains, USA, respectively)^18,19^. However, such interpretations need to be corroborated, as key processes involved in the formation and preservation of the filaments, particularly with respect to Py, remain unclear^20^. The validity and significance of any geobiological information gleaned from such records hinges on a robust understanding of the formation and preservation of morphological biosignatures^21^.

**Fig. 1:**
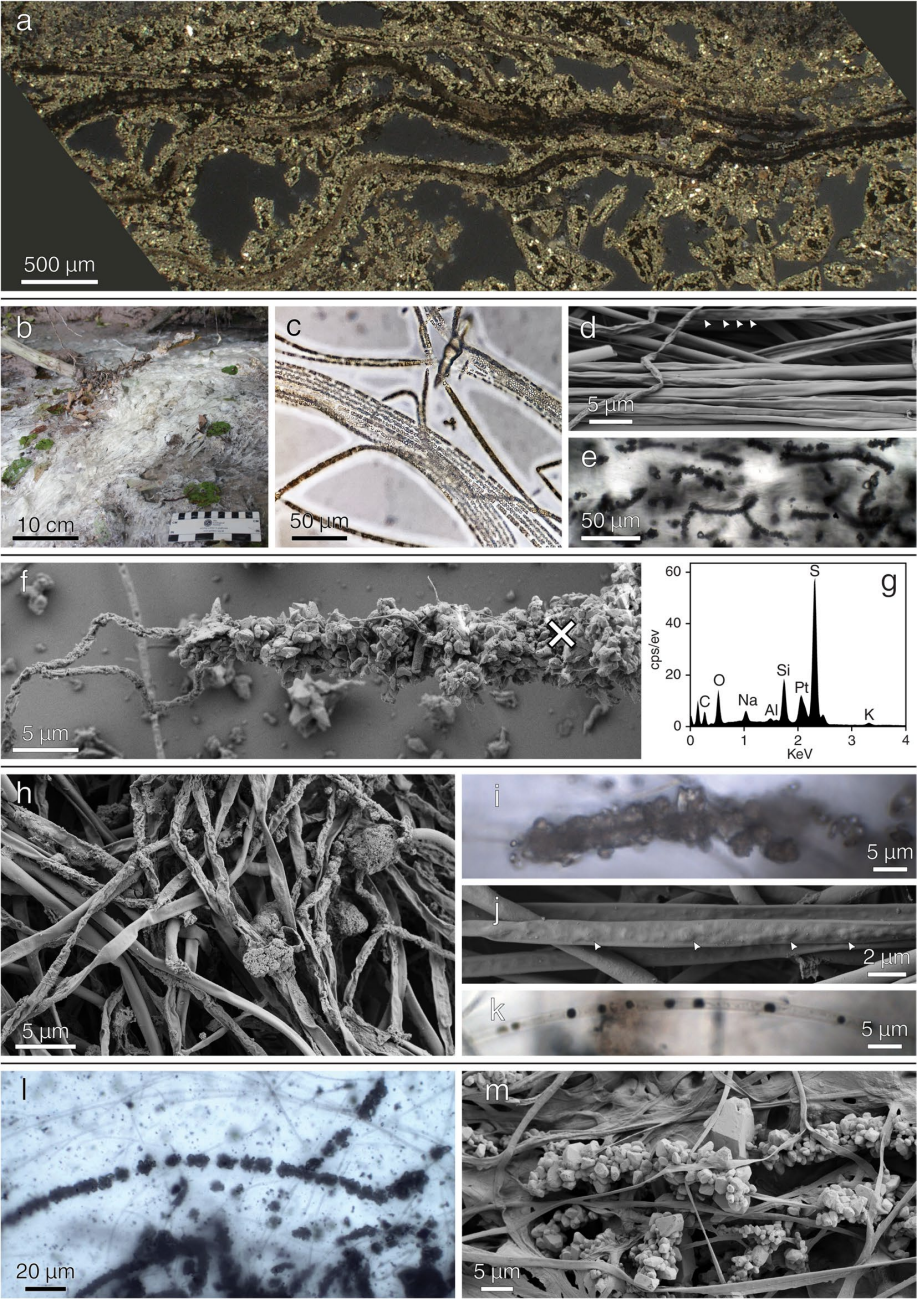
Key features of natural mats and experimental products. (**a**) Reflected light micrograph of pyritized filamentous structures of unknown origin in modern hydrothermal sulfide deposits (East Pacific Rise, RV Sonne research cruise SO62, 1989). (**b**) Field photo of the SOB-rich spring in Bad Alvaneu, Switzerland. (**c**–**d**) Transmitted light (**c**) and SEM micrographs (**d**) of the original SOB mats. The microbial mats are composed of bundles of ~1–2 μm wide filaments containing S⁰ inclusions (bright spherules in (**c**), arrows in (**d**)). (**e**) Transmitted light micrographs of the Mat-ASW experiments during 80 °C incubation. Filaments are preserved and some are encrusted with S⁰ released from cells. (**f–g**) SEM–EDX analysis of samples from Mats-Fhy experiments during 80 °C revealed the presence of crusts around individual filaments, consisting of S⁰ which was inferred from the detection of S and absence of Fe in EDX spectra; ×in (**f**) marks EDX measuring position. Note that the EDX data was truncated due to the lack of any peaks beyond 4 keV (**g**). (**h**–**k**) SEM and transmitted light micrographs of the Mat-FhyS experiments during 80 °C incubation. (**h**) Filaments vary from shriveled to smooth, partly covered by Fe–S minerals and (**i**) encrusted by released intracellular S⁰. Some filaments contained opaque globules of uncertain composition (**j**–**k**). (**l**–**m**) Transmitted light (**l**) and SEM (**m**) micrographs of S⁰ encrusted filaments observed in Mats-FhySSi experiments during 80 °C incubation. Note in (**l**) that the filaments are devoid of the S⁰ inclusions observed in case of the original mats (c–d) and some of the experiments (**j**–**k**).

Various authors described pyritic filamentous microfossils preserved from Archaean to Cambrian deposits and compared them with modern S⁰-cycling bacteria^16–19^. Furthermore, pyritized filamentous structures of unknown origin also occur in younger Phanerozoic rocks and modern hydrothermal sulfide deposits^22,23^ (Fig. 1a). Some of these ancient pyritized filaments indeed morphologically resemble those of modern S⁰-oxidizing bacteria (SOB), which are typically 1–2 μm in diameter (Fig. 1b–d). Modern filamentous SOB may occur within S⁰-rich mats in microoxic zones where oxygen (O_2_) and sulfide (“H_2_S", refers to total sulfide here) meet^24^. Although environments in which such conditions persist are quite diverse and range from deep sea hydrothermal systems to terrestrial sulfidic springs, their microbial communities tend to be very similar in terms of taxonomic composition, most commonly consisting of the ensheathed filamentous SOB genera *Thiothrix* and *Beggiatoa* (Table S1).

Here, we assessed the fate of filamentous SOB mats during early diagenesis (i.e., shallow burial conditions; ≤ 80 °C, ambient pressure) by conducting batch experiments with samples from a modern terrestrial sulfidic spring. By combining light microscopy, scanning electron microscopy coupled with energy-dispersive X-ray spectroscopy (SEM–EDX), UV–VIS spectrophotometry, micro-X-ray diffraction (μXRD), Fourier transform infrared (FTIR) and cryo-soft X-ray Tomography (cryo-SXT), we unraveled a sequence of early taphonomic key processes involved in the preservation of filamentous SOB mats. Finally, we discuss these findings in view of the identification, verification, and interpretation of potential morphological biosignatures preserved in Earth’s oldest rocks.

## Results and discussion

### Original SOB mats

**Fig. 2:**
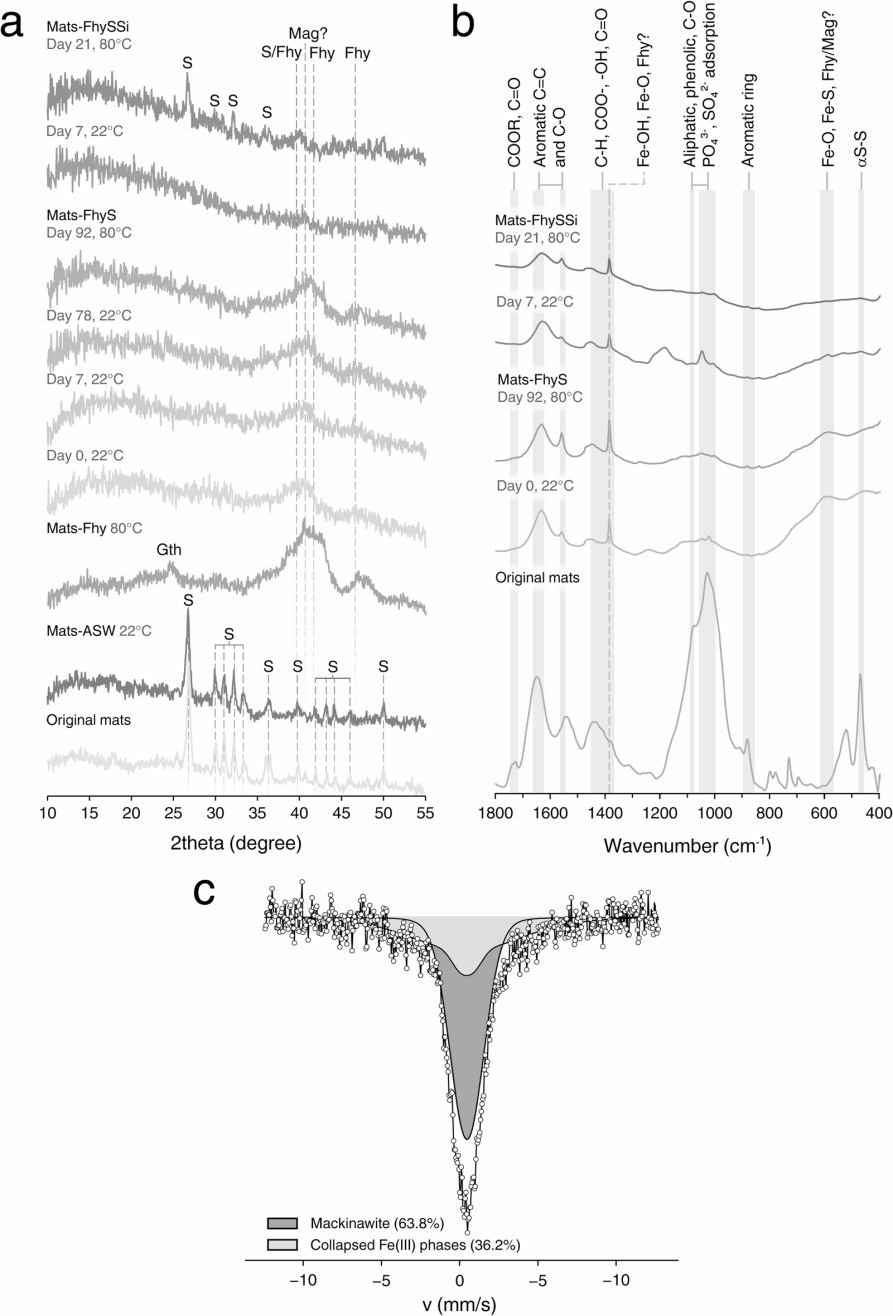
Mineralogical and organic analyses. (**a**) μXRD data (**b**) FTIR data for original mats and experimental products. (**c**) Mössbauer data for Mats-FhyS at the end of 80 °C experiment. S – sulfur, Fhy – ferrihydrite, Mag – magnetite, Gth – goethite.

Samples for the diagenesis experiment were collected from a sulfidic spring in Bad Alvaneu, Switzerland. The spring water was circumneutral and contained 70.4 µM and 0.3 µM of dissolved H_2_S and Fe (Supplementary Table 2), respectively, indicative of a sulfide-dominated system. Compared to other sulfidic springs in the area, the spring is relatively high in H_2_S and low in Fe (Supplementary Table 2). The sampled SOB mats consisted of up to several centimeter long filaments (Fig. 1b–d). Individual filaments contained S⁰ inclusions (0.5–2.0 µm in diameter), as evidenced by light microscopy, μXRD and FTIR (Figs. 1c, d and 2a, b). The filamentous morphology and presence of intracellular S⁰ granules are typical characteristics of *Thiothrix*, which comprises 6% of the total microbial mats community as shown by 16S sequencing (Supplementary Data sheet and Supplementary Fig. 1). Microbial communities in nearby springs (Zuelper and Arvadi) showed higher relative abundances of *Thiothrix* (14–17%^25^), reflecting site-specific biogeochemical variations.

The total carbon (C), nitrogen (N) and sulfur (S) contents of the mats were 10 wt.%, 1 wt.% and 57 wt.%, respectively. Notably, the amount of methanol-extractable S⁰ species was 0.9 wt.% and thus much lower than the total S content, indicating the presence of less reactive or extractable S species. μXRD analysis revealed the presence of elemental sulfur, while there was no evidence of Fe minerals (Fig. 2a). This is in good accordance with low amounts of acid (HCl- and HNO_3_)-extractable Fe (0.5 wt.%), suggesting low amounts of solid phase-associated Fe. FTIR analysis showed the presence of different organic bonds (especially related to carboxylates /–COOH–groups at 1650 cm^−1^), phosphates (PO_4_^3-^ at 1025 cm^−1^) and S⁰ (αS–S at 470 cm^-1^, Fig. 2b). Further interpretation of FTIR data can be found in Supplementary Discussion.

### Experimental design

Chemical parameters in the taphonomic experiment were adapted to either marine sediments or fluids in hydrothermal sulfide systems (see^26,27^). Four sets of experiments were performed with increasing complexity (I.–VI.; Table 1). First, the original mats were resuspended in artificial sea water (ASW) to evaluate potential effects of salinity on mats (“Mats-ASW”). Second, the original mats were incubated with 30 mM ferrihydrite (Fhy) in ASW to assess potential attachment of Fhy to the filaments (“Mats-Fhy”). Fhy, here used as a Fe source, is one of the most reactive Fe(III) minerals in nature, with an average concentration of 30 mM HCl-extractable Fe(III) in sediments^27^. Next, sulfide at a concentration of either 30 or 60 mM was added to ferrihydrite and the mats to investigate how FeS minerals could attach to and/or affect preservation potential of the filaments (“Mats-FhyS”). Finally, 5 mM dissolved silica was added to investigate how silica affects FeS mineral formation and the preservation of the filaments (“Mats-FhySSi”). In addition, some experiments were performed without mats but with Fe and S⁰ (with/without silica; “Biomorph” and “Biomorph Si”) to investigate for mineral biomorphs that may mimic biological morphology. The experiments were incubated sequentially at 22 °C and 80 °C for 7–78 days and 14 days, respectively, depending on the exact setup (see Methods).

**Table 1:** Overview of key experimental parameters.

Sampling method	Age of mats^1^	ASW (mL)	c (mmol/L)			Incubation *t* and T°C		Experimental	Final mineralogy	
			Fhy	S^2-^	Si^3^	22 °C	80 °C	Setup	Pyritization	Magnetism
Sacrificial	12 days	8	30	30	–	35 days	91 days	I. Mats-ASW	0%	−
								II. Mats- Fhy	2%	+
								III. Mats-FhyS	11% ± 4% (*n* = 2	+
Repetitive	> 1 year	80	30^2^	60^2^	5^3^	7 days	14 days	IV. Mats-FhySSi	3% ± 1% (*n* = 3)	−
								V. Biomorph	39% ± 2% (n = 2)	−
								VI. Biomorph Si	41% ± 1% (n = 2)	−

*V* Volume, *c* Concentration, *t* Time, *T*°C Temperature in degrees Celsius, *ASW* Artificial sea water, *Fhy* Ferrihydrite

^1^Since sampling date

^2^24 h equilibration after each addition

^3^Silica added just before starting the 80 °C incubation

### Initial taphonomy during storage and comparison to I. Mats-ASW

The color of microbial mats kept at 4 °C changed from white to shades of grey and black over 1–2 years, starting from the bottom of the container (Supplementary Fig. 2a). Microscopic observations showed that intracellular S⁰ was released from microbial cells and formed thin veneers coating the filaments (Supplementary Fig. 2b). The exact age of the mats used per experimental setup is shown in Table 1.

No clear difference was observed when mats were incubated in ASW at 22 °C versus the effect of long-terms storage in its native water. In fact, most of the intracellular S⁰ was released from the filaments within only a few days of 22 °C incubation in all experiments using 12-day old mats (Mats-ASW, Mats-Fhy and Mats-FhyS), probably due to cellular stress (Supplementary Fig. 3a, b, f). Upon release, S⁰ (indicated by μXRD, Fig. 2a) recrystallized in form of thin veneers coating the filaments (Supplementary Fig. 3a, b, g, h, i, j) and as dispersed particles, commonly crystallized in the form of rhombic dipyramids (Supplementary Fig. 3c, d).

After heating to 80 °C, the general filamentous structure of the mats was still intact, and the associated encrustations were still preserved (Fig. 1e). At the same time, yellowish colors of the liquid phases suggested the formation of polysulfides, perhaps due to the abiotic reduction of S⁰ by organic matter (OM) at elevated temperatures (Supplementary Fig. 2d). More specifically, H_2_S might have been oxidized coupled to the reduction of relatively oxidized biomolecules in the original mats (e.g., carboxylates), yielding secondarily formed S⁰ that further reacted with residual H_2_S to form polysulfides^28^.

### SOB mats and ferrihydrite in ASW (II. Mats-Fhy)

An early diagenetic encrustation of microbial filaments by Fe minerals could promote their preservation over geological time scales. Possible pathways include an attachment of Fe(III) nanoparticles to charged organic surfaces such as cell walls or extracellular polymeric substances (EPS)^29,30^. Indeed, the SOB mats were characterized by a negative surface charge (average zeta potential: − 4.3 ± 0.25 mV; *n* = 3 measurements), while the surfaces of Fhy were highly positively charged (average zeta potential: + 36.9 ± 0.42 mV; *n* = 3 measurements). Hence, an initial encrustation of the filaments by Fhy through electrostatic attachment would be possible.

At 22 °C (Supplementary Fig. 2e, Supplementary Movie), Fhy nanoparticles seemed to aggregate into bigger particles unrelated to microbial filaments, as indicated by light microscopy (Supplementary Fig. 3e). During the experiment, black magnetic minerals formed, as evidenced by tests with neodymium magnets (Supplementary Fig. 2f.). These minerals could be magnetite (Mag, Fe_3_O_4_) or greigite (Grg, Fe_3_S_4_). At 22 °C, Mag could form through microbial Fe(III) reduction, perhaps coupled to the oxidation of OM^31^. At 80 °C, however, Mag formation through Fe(III) reduction coupled to OM oxidation could also proceed abiotically^32^. Grg formation might be driven by H_2_S produced from abiotic reduction of S^0^ coupled to OM oxidation, as discussed earlier. However, there is no μXRD evidence for the presence of Grg. Furthermore, the only possible indication for Mag in experiments at 80 °C (wide reflection at 41.4 2θ) could also be due to the Fhy in the sample (presence indicated by double reflection around 46.9 and 47.9 2θ; Fig. 2a). Mössbauer spectroscopy could not resolve the mineral phase, detecting only collapsed Fe(III) phases that may represent different polycrystalline Fe(III) minerals in the system (Fig. 2c). In short, Fhy was partially transformed into a magnetic mineral, but S^0^ still seemed to be the primary mineral associated with the filaments, as inferred from the detection of S and absence of Fe in SEM–EDX data.

### SOB mats, ferrihydrite and sulfide in ASW (III. Mats-FhyS)

At 22 °C (Supplementary Fig. 2g), the Fhy turned immediately black upon the addition of 30 mM H_2_S, likely due to the formation of FeS nanominerals (Supplementary Movie)^33^. This reaction involves the reduction of Fhy by H_2_S (at pH 7.5 in form of the species HS^−^), first yielding Fe^2+^ and S^0^ (Eq. 1), followed by the subsequent reaction of the yielded Fe^2+^ with surplus HS^−^ to precipitate FeS (Eq. 2). The presence of FeS minerals such as mackinawite (Mkw, FeS) was not confirmed by μXRD or FTIR, perhaps due to the low crystallinity of the initial precipitates (Fig. 2a–b). Nonetheless, Mössbauer spectroscopy confirmed the presence of Mkw (Fig. 2c).

1 $${\text{2Fe}}\left( {{\text{OH}}} \right)_{{3}} + {\text{HS}}^{ - } + {5}{\text{H}}^{ + } \to {\text{ 2Fe}}^{{{2} + }} + {\text{S}}^{0} + {\text{6H}}_{{2}} {\text{O}}$$

2 $${\text{Fe}}^{{{2} + }} + {\text{HS}}^{ - } \to {\text{FeS}} + {\text{H}}^{ + } $$

OM signals from FTIR spectra of Mats-FhyS samples do not differ significantly from the original mats except in their peak intensities. Notably, some of the observed peaks (e.g. those indicative of COOH bonds, C–O bonds and PO_4_^3-^) were also found to be present in OM associated with Proterozoic microfossils^34,35^. FTIR was also inconclusive with regard to mineralogy. The broad peak at 600 cm^−1^ and sharp peak below 1400 cm^−1^ likely represent Fe–O(H) stretching and, in the case of the former, potentially reflect a mix of FeS and FeO^36,37^. Specifically, peaks characteristic for the Fe–O and Fe–OH bond stretching vibration are 585–610 cm^−1^ and below 900 cm^−1^ ^38,39^. Typically, low crystalline ferrihydrite has a strong and broad band with peaks at 440 cm^−1^ and 580 cm^−1^ ^40^. This peak could also overlap with the previously mentioned stretching vibration of Fe-S bonds found at 595 cm^−1^ ^41^.

**Fig. 3:**
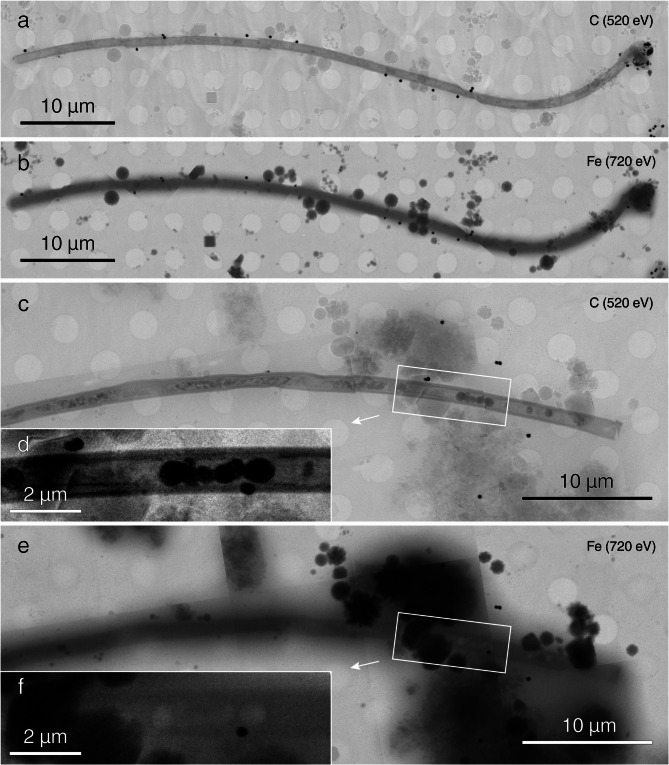
CryoSXT mapping of products from Mats-FhyS experiments. Darker color intensity denotes stronger signal in C or Fe edges (520 eV or 720 eV, respectively). Note that the filaments lack C on their surfaces (**a**) but are rich in Fe (**b**). In contrast, the inclusions are rich in carbon (**c–d**) and poor in iron (**e–f**). Arrows point to zoomed-in view of the highlighted regions in respective images.

Similar to the previous experiments, heating to 80 °C resulted in the formation of polysulfides, as evidenced by a yellow color of the solution (Supplementary Fig. 2h). Notably, the yellow color slowly dissipated after 4 °C storage for further analyses, indicating polysulfides were either not stable or the rate of production was slower than the rate of consumption (e.g., by organic matter sulfidation or pyrite formation) at lower temperatures. Furthermore, many filaments exhibited S⁰ encrustations at the end of 80 °C experiment, as observed with SEM and light microscopy (Fig. 1h–i), and some particles closely associated with the microbial mats were attracted by magnets (Supplementary Fig. 2h). While there is no μXRD evidence for the presence of magnetic minerals such as Grg or Mag (Fig. 2a), a FTIR peak at 589 cm^−1^ might indicate the presence of the latter^38,42,43^. Opaque intracellular globules were occasionally observed within the filaments after heating in this setup. Cryo-SXT revealed that the globules were not Fe minerals, but rather seemed to be enriched with OM, perhaps from residual membranes that hosted S^0^ or polyphosphates. Additionally, cryo-SXT showed conclusively thin Fe coatings on the surfaces of individual filaments (Fig. 3).

The observed Fe enrichments of filaments could be meaningful with regard to their fossilization potential, especially if they consist of (or potentially transfer to) geologically stable pyrite. However, sequential extraction indicated an exceptionally low pyritization extent during the first week at 22 °C (average Fe_HNO3_:Fe_total_ 0.9% ± 0.5; *n* = 6), followed by a slight increase after 78 days (average Fe_HNO3_:Fe_total_ 8.5% ± 1.4; *n* = 2). After heating at 80 °C, the pyritization extents were still relatively low and varied substantially (average Fe_HNO3_:Fe_total_ 10% ± 4.4; *n* = 6; note that the high error reflects variations in the experimental setups and sampling; see Supplementary Fig. 4 for detailed values per sampling point). Furthermore, Py was not detected by μXRD (Fig. 2a), and FTIR remained inconclusive as well (Fig. 2b). In case of FTIR, a small, broad peak below 400 cm^−1^ could be associated with Fe–S bands but cannot be considered robust considering the 350 cm^−1^ measuring limit^44–46^, while a broad peak between 400 and 600 cm^−1^ might result from unresolvable mixtures of phases with S–S, Fe–S and S–C bonds^47,48^. It should be noted, however, that the lack of pyrite detection via μXRD and FTIR might be due to low amounts and/or a low crystallinity of the precipitates.

### SOB mats, ferrihydrite, sulfide and silica in ASW (IV. Mats-FhySSi)

An early diagenetic silicification is widely considered beneficial for the preservation of morphological biosignatures^14,49^. To test for the potential influence of silica on the microbial mats investigated herein, we additionally performed Mats-FhyS experiments involving 5 mM silica (Mats-FhySSi). Silica was added only at 80 °C to prevent silica gel formation at lower temperatures, and 60 mM H_2_S were used to promote pyrite formation. The outcome of the Mats-FhyS and Mats-FhySSi experiments were fairly similar, with the filaments associated mainly with S⁰ veneers instead of Fe-S minerals, as observed by microscopy (Fig. 1l, m). Similar conclusions were also reached from μXRD and FTIR, in that the formed minerals were mostly amorphous and that the organic signals were reduced compared to the original mats (Fig. 2).

Further mineralogical analysis did not indicate the formation of magnetic minerals, perhaps due to the higher H_2_S concentrations in this experiment compared to the previous ones. This suggests that the “goldilocks zone” for magnetite/greigite formation depends on the relative proportions of Fe(III) minerals, Fe^2+^ release and H_2_S concentrations^27,50,51^.

Intriguingly, the pyritization extent in Mats-FhySSi (3 ± 0.9%, *n* = 3) was even lower than for the Mats-FhyS experiment (11 ± 4%) after 14 days of incubation at 80 °C (Table 1, Supplementary Fig. 4). In previous studies at similar pH, Fe/S ratios and comparable or lower Fe/S concentrations, pyritization extents were typically ≥ 40%^23,52,53^; hence, pyrite formation in our experiments may be limited by the mats and/or silica. At the same time, our experiments without mats and with/without added silica (see next section) also resulted in ~ 40% pyritization extent, which is comparable to those previous studies. Thus, our results indicated that (a) the presence of the mats themselves is a key factor inhibiting pyrite formation and (b) silica also contributed to inhibition of pyrite formation, but only in the presence of mats.

To explain the inhibition of pyrite formation, we need to consider the complex interactions between OM, Fe, S and silica. Inhibition effects of OM on pyrite formation are well known and diverse^27,53^. For instance, OM could protect FeS and S⁰ minerals from dissolution, thus reducing the formation of available polysulfides required to from Py. Specifically, OM decreases reactivity of FeS minerals by adsorbing onto them, which in turn limits FeS aggregation that may lead to Py transformation^53^. Furthermore, OM can react with H_2_S forming organically-bound S^27^. Meanwhile, silica can polymerize to form an amorphous silica gel coating, physically blocking access to reactive Fhy and FeS surfaces and preventing their further transformation^54^. Additionally, the formation of stable Fe-Si complexes may lower the availability of Fe for Py formation^55^. Silica may also modify the surface charge of the minerals and/or the mats, preventing mineral nucleation and growth^56^, and affect the interaction of Fe and S with metabolites/organic matrix released from mats during decay formation^57,58^. These processes plausibly explain why the filamentous structures were not associated with mineral phases that are known to be stable over geological time scales.

### Abiotic experiments (“Biomorphs”; V. FhyS and VI. FhySSi)

Abiotic processes may result in the formation of structures that might be confused with microbial filaments (“biomorphs”^4–7,59–61^). Abiotic experiments with and without silica (added at 80 °C, see above) showed high pyritization extents (average Fe_HNO3_:Fe_total_ 41% ± 1.3, *n* = 2 and 39% ± 1.7, *n* = 2, respectively: Table 1, Supplementary Fig. 2k–n; Supplementary Fig. 4), indicating for our experiments discussed before that OM indeed inhibits mineral (trans)formation processes to a certain extent^21,62,63^. As mentioned before, polymerization and complexation of silica can prevent further FeS transformation to Py^54,55^. The negligible difference in pyritization extents observed here indicated that silica alone did not affect pyrite formation.

In both experiments, the yielded minerals clustered together into spherules composed of euhedral crystals (3 µm ± 1, n = 32, Supplementary Fig. 3k–m). These resembled experimentally formed framboidal Py^64^ as well as framboidal-like and euhedral Py yielded by the sulfidation of biogenic and abiotic Mag, respectively^21,53^. SEM–EDX analysis indicated composition of these spherules to be FeS mineral, most likely Py (Supplementary Fig. 3n–o).

### Biosignature implications and next steps

A limited knowledge of microbial fossilization introduces uncertainties to the interpretation of ancient morphological biosignatures. For instance, hydrothermal sulfides from the 3.2 Ga Sulphur Springs Group in WA and the 1.4 Ga Gaobanhe deposit in China contain pyritic filaments of possible microbial origin^16,17^. However, the proposed microbial origin of these filaments requires further verification^20^. In both cases, microbiological interpretation in terms of metabolism is pending. Furthermore, pyritic filaments and rods in ~ 518 Ma and ~ 270 Ma non-hydrothermal deposits (Shuijingtuo Formation, China and Glass Mountains, USA) were interpreted as fossils of sulfate reducing microorganisms, possibly relatives of modern cable bacteria^18,19^. A better understanding of microbial taphonomy could lend support to such interpretations.

Our experiments demonstrated that SOB filaments morphologically remain stable under ambient pressure and shallow burial conditions typically persisting during early diagenesis in sulfur-rich environments, including those in the wider vicinity of hydrothermal sulfide systems (i.e., temperatures of 4–80 °C, high proximity to Fe and H_2_S concentrations of 30–60 mM). Notably, S⁰ released from SOB cells formed thin veneers coating individual filaments. Recently, it was shown that pyrite could form via replacement of initial S⁰ particles in cultures of Fe(III)- and S^0^-reducing bacteria, preserving the shape of the original S⁰ crust^65^. Although it appears plausible that such S⁰ potentially function as template promoting the precipitation of sulfur-bearing minerals, the FeS minerals formed in our experiments surprisingly were not directly associated with the mats, despite similar incubation conditions to the aforementioned cultures. In fact, we observed quite low pyritization rates in the presence of mats, probably due to the high OM content that inhibited rapid formation of Py^21,62,63^. Hence, the observed differences might be related to the ratio of live to dead microbes, community composition/abundance, chemical composition of microbial OM and/or the amount of EPS, but this needs to be further elucidated in future studies. Furthermore, multi-modal approach integrating morphology and mineralogy with geochemical analyzes of different environments would give significant weight to biogenicity of any putative microfossils^22^.

**Fig. 4:**
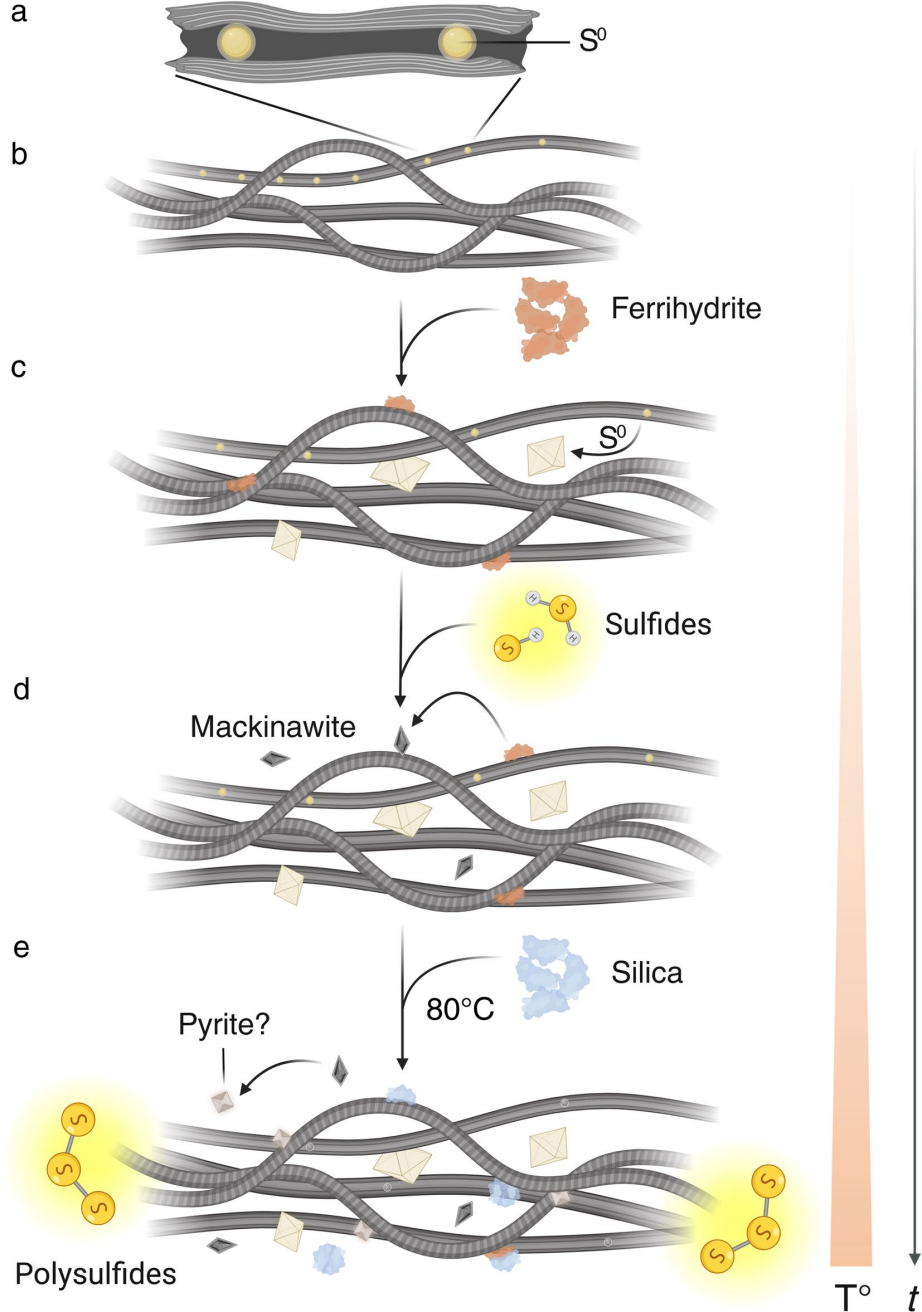
Proposed taphonomic sequence for filamentous SOB. (**a**–**b**) S⁰ globules inside the filaments. (**c**) Due to cellular stress, the intracellular S⁰ is released, forming extracellular coatings around individual filaments. The added ferrihydrite is loosely associated with the mats, mimicking interactions with Fe-containing minerals during burial. (**d**) Upon addition of sulfide, the ferrihydrite rapidly transforms into iron sulfides (FeS). (**e**) Addition of silica and its interaction with organic matter and Fe minerals which may affect their surface reactions. The hypothetical potential for pyritization upon exposure to high temperature is shown as accelerated by polysulfide formation. The figure was created with BioRender.com.

Nevertheless, even in the case of complete decomposition of cellular morphologies, the S⁰ encrustations retained the filamentous structure, albeit much thicker and potentially capable of further mineralization and growth (Fig. 4). Interestingly, the size range of the original filaments (~ 1–2 μm in diameter) is remarkably similar to that of the pyritic filaments observed in hydrothermal sulfides (i.e., the 3.2 Ga Sulphur Springs Group and the 1.4 Ga Gaobanhe deposit) and non-hydrothermal deposits (i.e., the ~ 518 Ma Shuijingtuo Formation and the ~ 270 Ma Glass Mountains)^16–19^. The higher thickness of individual filaments coated with S⁰ in our experiments (~ 6.0 μm) may seem significantly different, but in fact it is negligible considering that fossil filaments underwent compaction during burial. Regardless of the thickness, if ancient pyritic filaments such as those from the Sulphur Springs Group are indeed microbial in origin, the formation of metal sulfide crusts likely happened during later diagenetic stages. This may seem counterintuitive, as an early diagenetic formation of minerals is still widely considered critical for the preservation of delicate morphological biosignatures over geological time periods^23^. In order to test for plausibility, future studies might be paralleled by experimental tests, investigating the potential role of S⁰ templates for sulfide mineral formation. Finally, the impact of higher pressure and temperature on morphological biosignatures, as to be expected in some hydrothermal environments and/or during deeper burial diagenesis, needs to be experimentally tested.

## Methods

### Field site and sampling

The mats were collected from an outflow pipe of a S⁰-rich spring at Bad Alvaneu, Switzerland (Fig. 1b; coordinates: 46° 40′ 05.64″ N 9° 38′ 57.76″ E), either by scooping with gloved hands (ethanol-rinsed) or by aspiration into aseptic 50 mL syringes. The mats were stored in 1L Schott bottles at 4 °C. The total carbon (C), nitrogen (N) and sulfur (S) contents of dried mats were determined using the Euro Elemental analyzer, Hekatech, Germany. Microbial DNA was frozen and later extracted using a Qiagen PowerSoil Pro extraction kit and protocol. 16S microbial community analysis was performed using Illumina sequencing with 515F and 806R primers (see supplementary excel sheet; sequence read archive data was deposited to NCBI under BioProject accession number PRJNA1274659).

Water samples were filtered (mixed cellulose ester filters, 0.45 μm pore size) and divided into aliquots. Aliquots for metal and sulfide concentration measurements were then further processed (for metals: 1 M HCl, 800:200 μL sample; for sulfide: 1 M zinc acetate, 40:800 μL sample); aliquots for anions were not further treated. Ion concentrations were measured on diluted aliquots (with analytical grade 1% HNO_3_; Carl Roth) with inductively coupled plasma—mass spectrometry (ICP-MS, Agilent 7900, Agilent Technologies, USA) using helium (He) as carrier gas at a flow rate of 4 ml/min. Dissolved anions (F^−^ and SO_4_^2−^) were analyzed with ion chromatography (IC, 863, Metrohm, Germany). Nitrogen-containing ions (NH_4_^+^, NO_2_^−^ and NO_3_^−^) were measured using flow injection analysis (FIA, AA3, Seal analytical, UK). H_2_S was measured with the Cline assay (see “Geochemistry” section below). The obtained data is compiled in Table S2.

### Solution preparation for batch experiment

Artificial sea water (ASW) was prepared as matrix for the batch experiments using the following recipe: 17.30 g/L NaCl, 8.61 g/L MgCl_2_·6H_2_O, 0.99 g/L CaCl_2_·2H_2_O, 0.39 g/L KCl, 0.06 g/L KBr, 0.25 g/L NH_4_Cl, and 1.85 g/L NaHCO_3_. The pH of the resulting solution was adjusted to 7 using 0.5 mL of 1 M HCl, and the ASW was made anoxic by boiling and then purging with 50:50 N_2_:CO_2_ for 10 min per 100 mL solution.

2-line ferrihydrite (Fhy) was synthesized by dissolving 40 g Fe(NO_3_)_3_·9H_2_O in 500 mL ultrapure water (in the following referred to simply as “H_2_O”). 1 M KOH was used to adjust the pH of the solution to 7.1 via titration. After 2-h incubation at ~ 22 °C, the pH was readjusted to 7.5 and then the Fhy suspension was centrifuged (5000 × g; 10 min) and washed three times in H_2_O to remove nitrate ions. Finally, Fhy was resuspended in 200 mL H_2_O and purged with N_2_ for 30 min to remove O_2_.

Sulfide solutions (H_2_S) were freshly prepared for each experiment, using either sodium sulfide nonahydrate, Na_2_S·9H_2_O, Honeywell, CAS:1313–84-4 or sodium sulfide monohydrate, Na_2_S·H_2_O (Acros Organics, CAS:27610-45-3). The 0.5 M stock sulfide solutions (original pH > 10) were buffered with 2 M HEPES to pH ~ 7.5. Sulfide-HEPES solutions were made anoxic by purging with N₂ or dissolving in N_2_-purged water.

Silica solution was prepared from sodium metasilicate nonahydrate, Na_2_SiO_3_·9H_2_O, Sigma-Aldrich, CAS:13517-24-3. Stock silica solution was dissolved in N_2_-purged water and heated to 90 °C to ensure the breakdown of silicate polymers to monomeric species. The hot solution was subsequently added to samples maintained at 80 °C to achieve the final concentration. Upon dissolution, the dominant species are orthosilicic acid (Si(OH)_4_) and metasilicate anions (SiO_3_^2−^), collectively referred to here as “silica”. The initial solution is strongly alkaline; however, its addition did not significantly alter the experimental pH.

### Experimental setup

All sampling and fixation steps were done in a glovebox (MBraun Unilab Workstation, Germany) under a N_2_ atmosphere (O_2_ < 0.1–50 ppm). Approximately 1 cm^2^ of wet mats (average wet weight 350 mg) was used per sampling point in each bottle. Sacrificial setups (Mats-ASW, Mats-Fhy and Mats-FhyS) contained one piece of mats per bottle, while repetitive setups (Mats-FhySSi) contained multiple pieces of mats (~ 5) corresponding to the number of sampling points planned. It must be noted that homogenizing the mats is rather difficult. Hence, the reported amounts of mats are approximate due to their heterogeneity. After weighing the mats into the serum vials, ASW, Fhy and finally sulfide-HEPES were sequentially added. In case of biomorph experiments (Biomorph and Biomorph Si), no mats were used.

In the sacrificial setups, all the components were added directly one after another. The Mats-ASW, Mats-Fhy and Mats-FhyS incubation at 22 °C and 80 °C lasted for 78 and 14 days, respectively. In the repetitive setups, a 24-h equilibration time was allowed between additions. For the Mats-FhySSi and both biomorph experiments, incubations at 22 °C and 80 °C lasted for 7 and 14 days, respectively. Sampling for aqueous geochemical analyses was performed every 3–7 days, while samples for mineralogical and solid phase analyses were taken before increasing the incubation temperature and at the end of experiments. For a detailed overview of the setups see Table 1.

### Geochemistry

Solid particles were removed from the aqueous phase by centrifugation at 13,400 g for 5 min and subsequently filtered through a 0.45 μm mixed cellulose ester (MCE) filter. It was visually observed that some colloidal nanoparticles could not be centrifuged and filtered, especially during early stages of experiments at 22 °C. We suspect that these particles were nanoparticulate FeS that might contribute to an overestimation of dissolved Fe and H_2_S. Aqueous phase was analyzed for dissolved Fe^2+^, total sulfide (H_2_S), and sulfate (SO_4_^2−^). Supernatant of samples for dissolved Fe^2+^ concentrations were acidified with 1 M HCl and quantified spectrophotometrically using the Ferrozine assay^66^. S⁰ was extracted from the mats using methanol (100 mg mats per 10 mL methanol) and measured by high-performance liquid chromatography (HPLC, class VP with RID 10 A and DAD 457 SPD M 10A VP detectors, Shimadzu Prominence, Japan; ReproSil-Pur 200 ODS-3 column 250 · 4 mm, 5 µm, Dr. Maisch GmbH, Germany) after 20 min retention time. Sulfide concentrations were quantified spectrophotometrically using the modified Cline assay^67^. Polysulfides in the aqueous phase were observed visually via a yellowish coloration. Sulfate concentrations were determined by a turbidimetric protocol via barium sulfate formation. 100 μL of samples were mixed with 20 μL of conditioning reagent (30 ml H_2_O, 3 ml concentrated HCl, 7.5 g NaCl, 10 ml 95% ethanol and 5 ml glycerol) and 30 μL of BaCl_2_ in a 96-well plate, and the turbidity was measured via absorbance at 420 nm after 5 min.

### Mineralogy

After separation from the liquid phase, solid phases (i.e. microbial mats and minerals) were washed 3 times with H_2_O to remove residual salts. Sequential iron extraction of solid phases was performed using 6 M HCl (for reactive Fe minerals: magnetite, Mag, Fe_3_O_4_; mackinawite, Mkw, FeS; greigite, Grg, Fe_3_S_4_) and 8 M HNO_3_ (for Py) to determine the extent of pyritization over time^68^. Extraction with 6 M HCl was conducted for 24 h in an anoxic chamber in the presence of Ti(III)-citrate to prevent oxidation of dissolved sulfide to S⁰, which could reduce Fe extraction yields^69^. Solid residues from the 6 M HCl extraction step were washed with 1 M HCl and subsequently extracted with 8 M HNO_3_ for > 2 h. Total iron concentrations in the extracts were quantified spectrophotometrically via the ferrozine assay using hydroxylamine as the reducing agent^66^.

For μXRD and FTIR, samples were dried in Eppendorf tubes and stored in anoxic bottles under a N_2_ atmosphere until analysis. μXRD was performed using a Bruker’s D8 Discover GADDS XRD2 micro-diffractometer equipped with a standard sealed tube with a Co-anode (Co Kαradiation, λ = 0.179 nm) at 30 kV/30 mA. The total measurement time was 240 s at two detector positions (15◦ and 40◦). Phase identification was validated using Match! software for phase identification from powder diffraction (Match! Crystal Impact, Germany, version 3.15 278) with the Crystallography Open Database (COD-Inorg REV211633 2018.19.25). FTIR was performed using Vertex 80v FTIR spectrometer (Bruker, USA). Dried samples were diluted with KBr for analysis (Carl Roth, USA) and pressed into 250 mg pellets. Due to differences in sample opaqueness and signal saturation, different KBr:Sample ratios were used per sample type and those with optimal signal were used for further analysis (750 mg KBr:4.5–6.5 mg sample). Spectra were scanned 64 times per sample, from 370 to 4500 cm^−1^ (4 cm^−1^ resolution), averaged, and normalized (min: 0, max: 1) to obtain the final spectra.

^57^Fe Mössbauer spectroscopy analysis was applied for the identification of magnetic minerals present in the samples. Sample preparation was performed in the glovebox under anoxic conditions. Magnetic particles were separated from the sample using neodymium (Nd) magnets and a pipette, and then filtered using a 0.22 µm MCE filter and wrapped with O_2_-impermeable Kapton® tape. Prior to analysis, the samples were stored at − 20 °C. Samples were placed in a closed-cycle exchange gas cryostat (Janis Research, USA) under He backflow to minimize air exposure during the measurements. Mössbauer spectra acquired at 140 K with a constant acceleration drive system (WissEL, Germany) in transmission mode by using a ^57^Co/Rh source. Spectral calibration was carried out using a 7 µm thick ^57^Fe reference foil measured at room temperature. Spectral fitting was performed with Voigt Based Fitting (VBF) routine^70^ in Recoil software (University of Ottawa) by constraining half width at half maximum (HWHM) as 0.121 mm s^-1^ (details in supplementary Table S3).

### Microscopy

Transmitted light microscopy was performed with a Zeiss Axioskop 2 plus microscope coupled with Euromex CMEX 5 camera. Reflected light microscopy was performed using Keyence microscope with VH-ZST objectives (Keyence, Japan). The samples were observed rapidly under the microscopy to prevent drying. Original light micrographs were processed using Adobe Photoshop 2022’s auto tone, contrast, and color scripts; each image was visually cross-checked against the live microscope view to ensure that no color shift or other artefacts had been introduced. The final micrograph plates were assembled in Adobe Illustrator 2022. Certain micrographs were photographed on different planes of focus and were subsequently aligned and stacked using Zerene Stacker 1.04.

Morphological analysis was carried out using a Crossbeam 550L scanning electron microscope (SEM; Zeiss, Oberkochen, Germany) operated at an acceleration voltage of 2 kV and a working distance of 5 mm. All micrographs were acquired with the Secondary Electron Secondary Ion (SESI) detector. Elemental composition was determined by energy-dispersive X-ray spectroscopy (EDX) using the same Zeiss Crossbeam 550L SEM, equipped with an Oxford Instruments Energy Dispersive Spectrometer (UltimMax 100, Oxford Instruments, Abingdon, United Kingdom). EDX point analyses were conducted at an acceleration voltage of 10 and 20 kV and a probe current of 1.5–2 nA, with a working distance of 5 mm and a detector deadtime of about 30–40%. Two different sample preparation protocols were used for SEM–EDX analysis, one for preserving the cellular structure and the other for mineral preservation as detailed in^67^. The EDX data was truncated due to the lack of any significant peaks beyond 4 keV.

### Surface charge

Surface charge of solid materials was determined via a Zetasizer Nano instrument. For the original mats, H_2_O was used as the matrix for ~ 1 cm^2^ of material. For Fhy measurements, 30 mM were suspended in ASW. The voltage, attenuation and the number of runs were automatically controlled by the instrument. The measured electrophoretic mobility was converted to surface charge (Zeta potential) using the Smoluchowski method^71^. Measurements were performed in triplicate and averaged.

### Cryo-soft X-ray Tomography (cryo-SXT)

Cryo-SXT was performed at B24 beamline of Diamond Light Source in Harwell Science and Innovation Campus (Oxfordshire, UK) to capture images of cellular architecture up to 25–40 nm resolution. Samples from Mats-ASW, Mats-Fhy and Mats-FhyS setups were imaged. Transmission electron microscopy grids (TEM, Quantifoil R 2/2 on 200 gold mesh) were used as the sample carriers. The grids were washed by plasma glow using PELCO easiGlow cryoEM glow discharge unit (Agar Scientific, product code: AGB7361) to render them hydrophilic. Due to the viscous nature of aggregated filaments, isolating singular filaments was challenging. A specific sample preparation method was used to “fish out” the filaments. A piece of parafilm (2 cm^2^) was laid flat onto a surface with a black background for a better contrast with the white filaments. A drop of 30–100 μL of filaments was placed onto the parafilm and gently separated using forceps. Then, the TEM grid was held with an inverted forceps and dipped into the sample like a fishing net. Excess water was removed by blotting onto a filter paper after applying 250 nm gold nanoparticles, which were added to the samples as image registration markers. Afterwards, the sample grids were cryopreserved into liquid nitrogen-cooled liquid ethane using Leica EM GP plunge freezer, which allows for vitrification of the sample, avoiding crystalline ice formation to better preserve cellular ultrastructure. Cryopreserved samples, arranged in sets of four, were placed under high vacuum conditions (10^−8^ Torr) into the transmission X-ray microscope (UltraXRM-S220C, Carl Zeiss X-ray Microscopy Inc.). First, lower resolution visible light mosaics were assembled for each sample to verify the alignment of each grid and to identify regions of interest (ROIs). Subsequently, for each ROI, a 2D X-ray mosaic was mapped at the C-edge (520 eV) and the Fe-edge (720 eV).

## Supplementary Information

Supplementary Information 1.

Supplementary Information 2.

Supplementary Video 1.

## Data Availability

Source data are provided with this paper and its supplementary information files.
