# Supplementary material for: Experimental diagenesis reveals preservation of biosignatures in filamentous sulfur mats under hydrothermal conditions
Source: Sci Rep. 2025 Oct 31;15:38114. doi: 10.1038/s41598-025-25172-4 (PMC12578821; doi:10.1038/s41598-025-25172-4)
Supplement: Supplementary file 2 — Supplementary Information 2. [file 41598_2025_25172_MOESM2_ESM.pdf]

Experimental diagenesis reveals preservation of biosignatures in filamentous sulfur mats under hydrothermal conditions

Hrvoje Višić[1], Jan-Peter Duda[2], Stefan Fischer[3], Cristina Escudero[1], Fatih Sekerci[1], Andreas Kappler[1,4], Muammar Mansor[1]*

[1] Department of Geosciences, University of Tübingen, Tübingen, Germany

[2] Department of Geobiology, Geoscience Center, University of Göttingen, Göttingen, Germany

[3] Tübingen Structural Microscopy Core Facility, University of Tübingen, Tübingen, Germany

[4] Cluster of Excellence EXC 2124, Controlling Microbes to Fight Infection, University of Tübingen, Tübingen, Germany

*Corresponding author: muammar.mansor@uni-tuebingen.de

Supplementary Information

Supplementary Tables 1–3

Supplementary Figures 1–4

Supplementary Discussion

Supplementary Table 1. Overview of microbial mats formed by similar taxa as those used in the experiments, but occurring in different environments.

Major taxon	Environment	Location	Reference
Thiothrix	Hydrothermal vents	Cape Palinuro, Italy	Mattison et al. 1998[1]
	Sulfidic cave systems	Lower Kane Cave, Wyoming, USA	Engel et al. 2004[2]
		Frasassi cave, Italy	Macalady et al. 2006[3]
	Brine pools	Panarea Aeolian Archipelago, Italy	Gugliandolo et al., 2015[4]
	Geothermal hotsprings	Continental Croatia	Kostešić et al., 2023[5]
	Cold sulfidic springs	Western Carpathian, Slovakia	Nosalova et al., 2023[6]
Beggiatoa	Hydrothermal vents	Cape Palinuro, Italy	Mattison et al. 1998[1]
	Sulfidic cave systems	Lower Kane Cave, Wyoming, USA	Engel et al. 2004[2]
		Frasassi cave, Italy	Macalady et al. 2006[3]
	Brine pools	Panarea Aeolian Archipelago, Italy	Gugliandolo et al., 2015[4]
Sulfurovum	Sulfidic cave systems	Frasassi cave, Italy	Macalady et al. 2006[3]
	Hydrothermal vents	San Pedro, California, USA	Miranda et al., 2016[7]
	Geothermal hotsprings	Continental Croatia	Kostešić et al., 2023[5]
Arcobacter	Sulfidic cave systems	Frasassi cave, Italy	Macalady et al. 2006[3]
	Cold sulfidic seeps	Nile Deep Sea Fan, Mediterranean	Grünke et al., 2011[8]
	Coastal sediments	Mramornaya Bay, Crimea	Pimenov et al., 2018[9]
Halothiobacillaceae	Geothermal hotsprings	Continental Croatia	Kostešić et al., 2023[5]
	Cold sulfidic seeps	Western Carpathian, Slovakia	Nosalova et al., 2023[6]
Thiomargarita	Cold sulfidic seeps	Nile Deep Sea Fan, Mediterranean	Grünke et al., 2011[8]
Desulfuromusa	Hydrothermal vents	San Pedro, California, USA	Miranda et al., 2016[7]
Desulfuromonas	Coastal sediments	Mramornaya Bay, Crimea	Pimenov et al., 2018[9]

Supplementary Table 2. Geochemistry of the water at the sampled spring compared to nearby springs from other references.

		Bad Alvaneu*	Zuelper[10]	Arvadi[11]	Arvadi[12]
mM	SO_4^{2-}	7.3	12.6	8.4	8.3
	Ca	6.5	10	7	
	Mg	2.7	5.3	3	
µM	S^{2-}	70.4			2.5
	F^-	48.1		83.4	
	K	23.0	30.7	27.3	
	NH_4^+	5.0		2	
	NO_3^-	1.15	18.3	0.1	
	Fe	0.3		2.5	17.2
	Mn	0.15			
	Ba	0.1			
	Mo	0.02			
	As	0.01			
	U	0.005			
	Se	0.005			
	Cr	0.004			
	Ni	0.003			
	V	0.003			
	Ag	0.002			
	Co	0.002			
	Cu	0.001			

*This study

Supplementary Table 3. Parameters used for Mössbauer spectroscopy analysis. CS: center shift, QS/ε: quadrupole splitting, H (T): Hyperfine field, χ^2: goodness of fit.

T (K)	Site	CS (mm/s)	QS/ε (mm/s)	H (T)	Relative area (%)	χ^2
140 K	Mackinawite	0.49	0.03	-	63.8	0.57
	Collapsed Fe(III) phase	0.45	0.00	18.07	36.2	

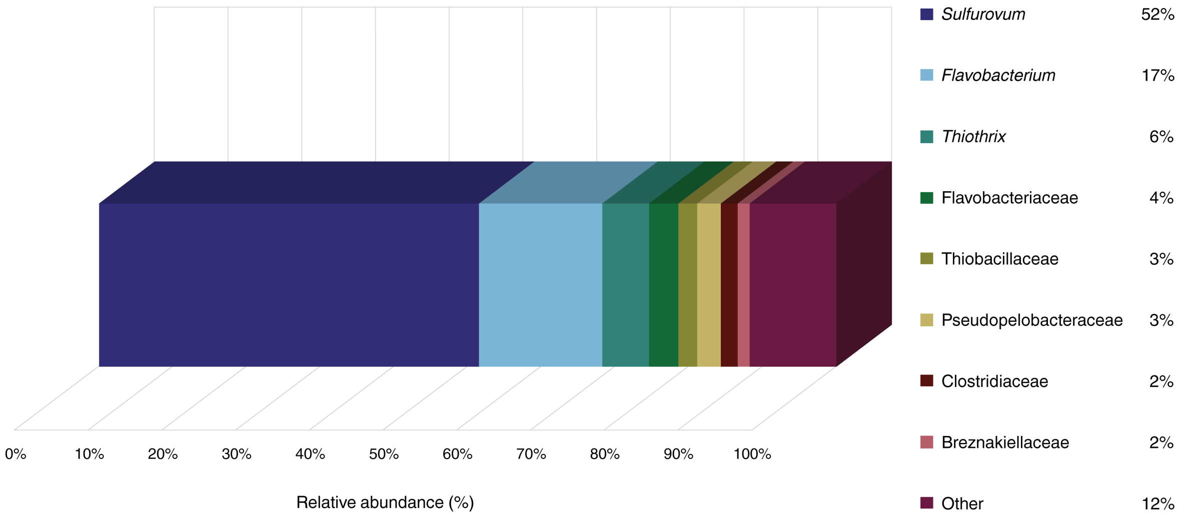

Supplementary Figure 1. 16S-based microbial community composition. The category “Other” includes all taxa that was <1% in relative abundance.

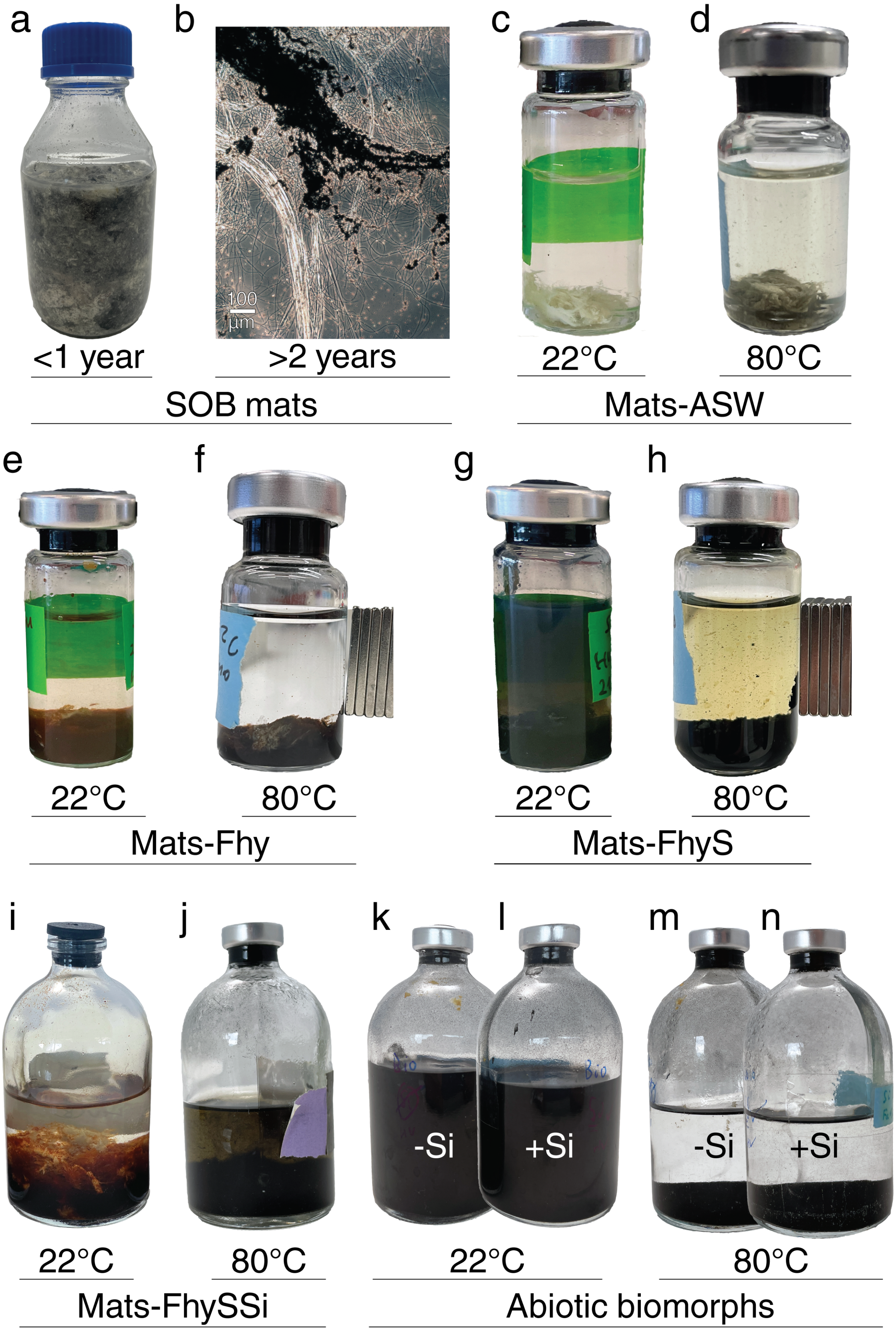

Supplementary Figure 2. Photographs of the incubation experiments at different stages (not to scale). (a) Mats after a few months of storage at 4°C in a 1 L Schott bottle, **(b)** Mats after more than 2 years in storage. Filamentous morphology is retained, but some mats are

encrusted with S^o (opaque particles). Intracellular S^o globules are no longer observed. **(c–j)** Samples at the end of the incubation at 22°C or 80°C . **(c–d)** Mats-ASW. **(e–f)** Mats-Fhy. **(G–h)** Mats-FhyS. **(i–j)** Mats-FhySSi. (**k–n)** – Biomorph experiments without (k, m) and with Si (l, n) after 22°C or 80°C incubations. Neodymium magnets are visible on the right side of the vials if magnetism was observed. Yellow polysulfides are visible in d, g, h and j.

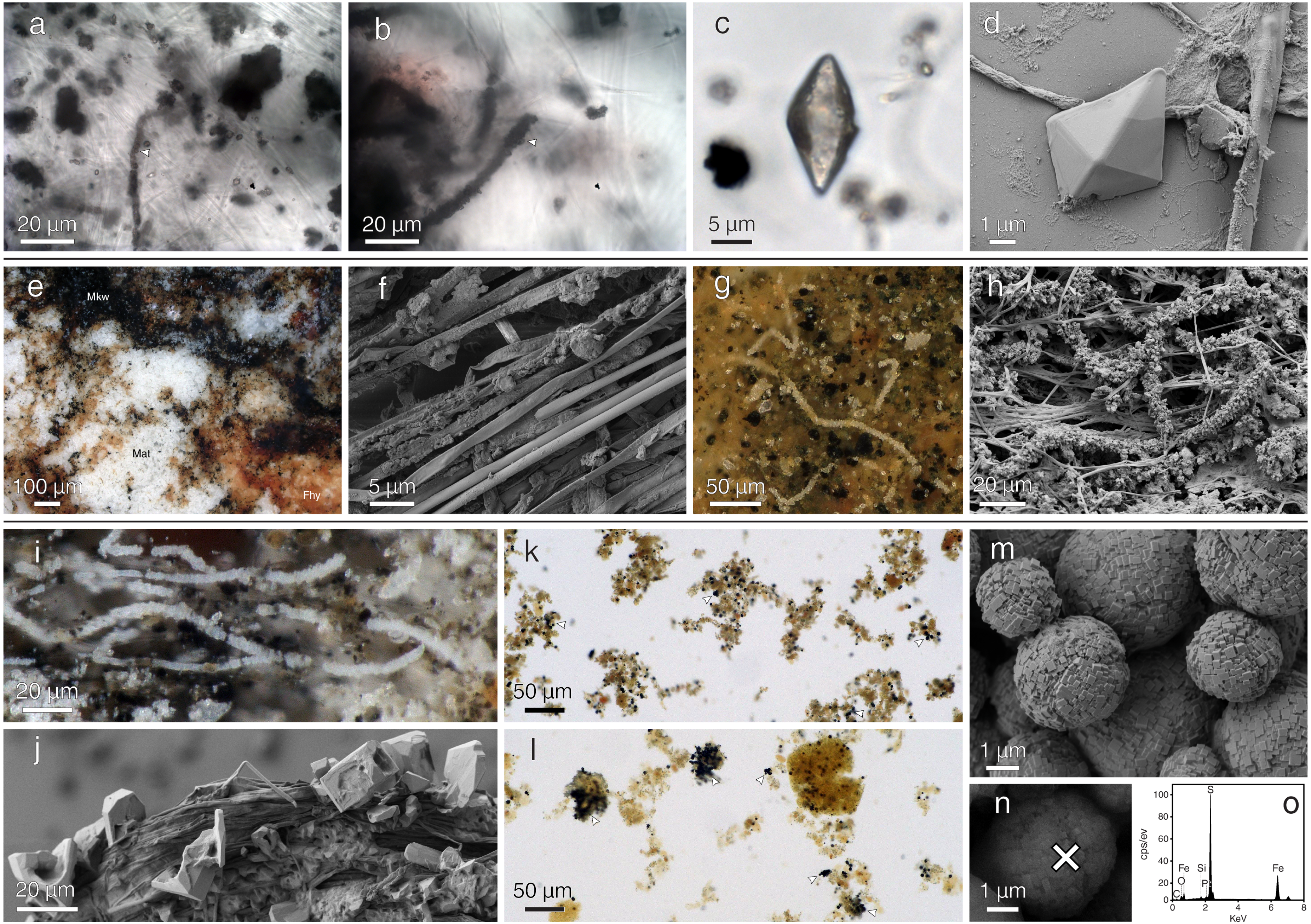

a
20 µm
b
20 µm
c
5 µm
d
1 µm
e
Mkw
Mat
Fhy
100 µm
f
5 µm
g
50 µm
h
20 µm
i
20 µm
j
20 µm
k
50 µm
l
50 µm
m
1 µm
n
1 µm
o
cps/ev
KeV
S
Fe
Si
O
C
P

Supplementary Figure 3. Photo plate of samples taken during different phases of the incubation experiments. (a–b) Transmitted light micrographs of Mat-ASW (a) and Mat-Fhy (b) after 22°C incubation shows Sº encrustation of some filaments (white arrowheads). Hairlike strands of non-encrusted filaments are barely visible in the background as thin grey lines. Note clustering of orange Fhy minerals in the background in (b). **(c–d)** Micrographs of recrystallized rhombic dipyramidal Sº minerals observed in Mat-ASW under transmitted light (c) and in Mat-Fhy using SEM (d). **(e–f)** Observed heterogeneity of Mats-FhyS during 22°C visible after 3 days under reflected light (e) and after 11 days using SEM (f). Some filaments reveal a smooth surface, others are slightly covered or encrusted by Fe-S minerals. Fhy – ferrihydrite, Mkw – mackinawite. **(g–j)** Reflected light (g, i) and SEM micrographs (h, j) of visible Sº encrustation on the filaments in Mat-FhyS (i–j) and Mat-FhySSi (g–h). **(k–l)** Reflected light micrographs of biomorph experiments without Si (k) and with Si (l) after 80°C and 21 days. Note clustering of FeS minerals into framboid-like structures (white arrowheads). **(m)** SEM micrographs of framboid-like spherules from biomorph setup without Si at the end of the experiment. **(n–o)** SEM-EDX analysis. Spectral graph (o) indicates spherules (n) are composed of FeS, most likely pyrite; × in (n) marks EDX measuring position.

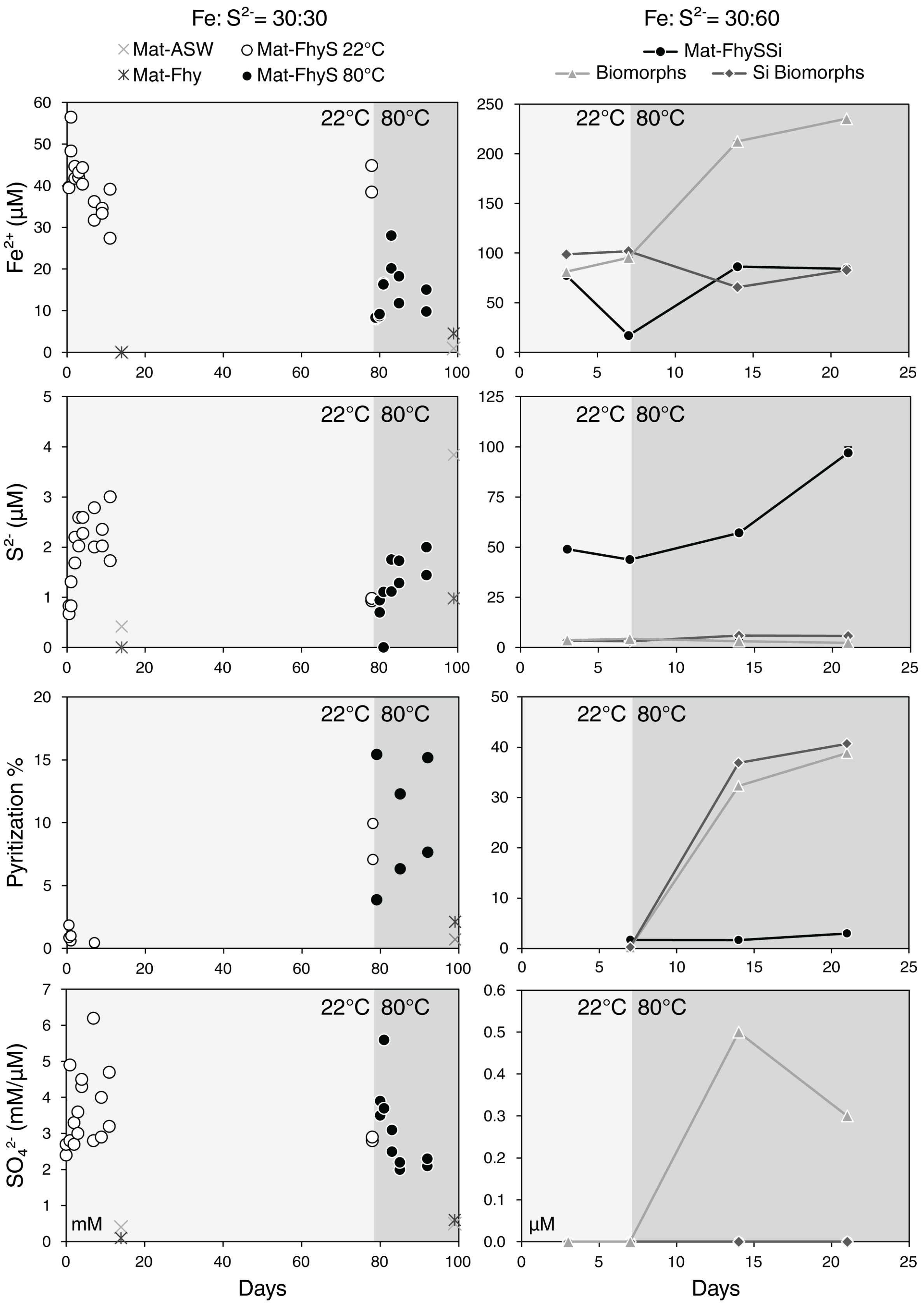

Supplementary Figure 4. Dissolved species concentrations per experimental setup. Fe^{2+}, S^{2-}, pyritization percent and SO_4^{2-} are shown per rows for each setup. Light grey area

signifies 22°C incubation phase, while dark grey area signifies 80°C incubation. Note that SO_4^{2-} concentrations reached ~6 mM in the first set of experiments (fresh mats, 12 days old), compared to negligible values in the 2nd set of experiments (old mats, > 1 year). This could be attributed to active microbial S^0 oxidation in the former.

Supplementary Discussion: FTIR interpretation

Based on the FTIR of the original mats (**Fig. 2b**), the peak around 1,750 cm^{-1} could correspond to C=O stretching in fatty acids and esters[13] or COOH/COOR stretching in carboxylic acids and aromatic esters[14]. Peak around 1,650 cm^{-1} could be attributed to stretching of aromatic C=C or asymmetric C–O stretching in carboxylates (COO−), which is furthermore supported by the peak around 1440 cm^{-1} where both OH deformation and symmetric C=O stretching from COOH or other carboxylic acid structures[15] could be seen. The strongest peak is visible around the 1025 cm^{-1} which we attribute to nucleic acid phosphates[16]. Another possibility for the origin of P could be storage of polyphosphate granules inside SOB mats[17,18]. A smaller peak at 880 cm^{-1} could be interpreted as aromatic ring stretching[14]. Finally, the sharp peak at 470 cm^{-1} is attributed to αS–S stretching in S_8 [19,20].

After experimental treatment, the most prominent organic peaks are still visible albeit much smaller in size. In addition, numerous poorly defined broad peaks are observed that could correspond to their organic or mineral signals. Note that intensities between samples are not directly comparable as the samples were treated differently for analyses depending on the optimal signal saturation. Nevertheless, trends are visible when comparing between the 22°C and 80°C samples.

The sharp S–S bond signal disappears after experimental treatment, replaced by broad peaks between 400–600 cm^{-1} that could be attributed to either Mag at 589 cm^{-1} or ferrihydrite at 585 cm^{-1} [21–23]. It is important that S^0 was not consumed, but rather the signal was likely diluted by the Fe minerals. Around 440 cm^{-1}, it is possible to have signals from Fe–S, S–S and S–C bonds[24,25]. There is small, broad peak below 400 cm^{-1} that could also be

associated with Fe–S bands but this signal is undiagnostic due to the set measuring limit of 350 cm^{-1} [26–28].

Supplementary References

1. Mattison, R. G. *et al.* Chemoautotrophic Microbial Mats in Submarine Caves with Hydrothermal Sulphidic Springs at Cape Palinuro, Italy. *Microb. Ecol.* **35**, 58–71 (1998).

2. Engel, A. S., Porter, M. L., Stern, L. A., Quinlan, S. & Bennett, P. C. Bacterial diversity and ecosystem function of filamentous microbial mats from aphotic (cave) sulfidic springs dominated by chemolithoautotrophic "Epsilonproteobacteria". *FEMS Microbiol. Ecol.* **51**, 31–53 (2004).

3. Macalady, J. L. *et al.* Dominant Microbial Populations in Limestone-Corroding Stream Biofilms, Frasassi Cave System, Italy. *Appl. Environ. Microbiol.* **72**, 5596–5609 (2006).

4. Gugliandolo, C. *et al.* Changes in prokaryotic community composition accompanying a pronounced temperature shift of a shallow marine thermal brine pool (Panarea Island, Italy). *Extremophiles* **19**, 547–559 (2015).

5. Kostešić, E. *et al.* Microbial Diversity and Activity of Biofilms from Geothermal Springs in Croatia. *Microb. Ecol.* **86**, 2305–2319 (2023).

6. Nosalova, L., Mekadim, C., Mrazek, J. & Pristas, P. Thiothrix and Sulfurovum genera dominate bacterial mats in Slovak cold sulfur springs. *Environ. Microbiome* **18**, 72 (2023).

7. Miranda, P. J., McLain, N. K., Hatzenpichler, R., Orphan, V. J. & Dillon, J. G. Characterization of Chemosynthetic Microbial Mats Associated with Intertidal Hydrothermal Sulfur Vents in White Point, San Pedro, CA, USA. *Front. Microbiol.* **7**, (2016).

8. Grünke, S. *et al.* Niche differentiation among mat-forming, sulfide-oxidizing bacteria at cold seeps of the Nile Deep Sea Fan (Eastern Mediterranean Sea): Niche differentiation among sulfide oxidizers. *Geobiology* **9**, 330–348 (2011).

9. Pimenov, N. V. *et al.* Structure of Microbial Mats in the Mramornaya Bay (Crimea) Coastal Areas. *Microbiology* **87**, 681–691 (2018).

10. Strauss, H. *et al.* Multiple sulphur and oxygen isotopes reveal microbial sulphur cycling in spring waters in the Lower Engadin, Switzerland. *Isotopes Environ. Health Stud.* **52**, 75–93 (2015).

11. St Clair, B., Pottenger, J., Debes, R., Hanselmann, K. & Shock, E. Distinguishing Biotic and Abiotic Iron Oxidation at Low Temperatures. *ACS Earth Space Chem.* **3**, 905–921 (2019).

12. Koeksoy, E. *et al.* A case study for late Archean and Proterozoic biogeochemical iron- and sulphur cycling in a modern habitat—the Arvadi Spring. *Geobiology* **16**, 353–368 (2018).

13. Schmitt, J. & Flemming, H.-C. FTIR-spectroscopy in microbial and material analysis. *Int. Biodeterior. Biodegrad.* **41**, 1–11 (1998).

14. Artz, R. R. E. *et al.* FTIR spectroscopy can be used as a screening tool for organic matter quality in regenerating cutover peatlands. *Soil Biol. Biochem.* **40**, 515–527 (2008).

15. Thomas, G. J. *Applications of Infrared Spectroscopy in Biochemistry, Biology, and Medicine.* Frank S. Parker. *Q. Rev. Biol.* **47**, 477–477 (1972).

16. Orhan-Yanıkan, E., Gülseren, G. & Ayhan, K. Protein profile of bacterial extracellular polymeric substance by Fourier transform infrared spectroscopy. *Microchem. J.* **156**, 104831 (2020).

17. Langer, S. Polyphosphate in marine environements and Beggiatoa sp. (2019) doi:10.18453/ROSDOK_ID00002582.

18. Geerlings, N. M. J. *et al.* Polyphosphate Dynamics in Cable Bacteria. *Front. Microbiol.* **13**, 883807 (2022).

19. Meyer, B. Elemental sulfur. *Chem. Rev.* **76**, 367–388 (1976).

20. Steudel, R. & Eckert, B. Solid Sulfur Allotropes. in *Elemental Sulfur and Sulfur-Rich Compounds I* (ed. Steudel, R.) vol. 230 1–80 (Springer Berlin Heidelberg, Berlin, Heidelberg, 2003).

21. *Iron Oxides in the Laboratary*. (Wiley-VCH Verlag GmbH, Weinheim, Germany, 2000). doi:10.1002/9783527613229.

22. Mazzetti, L. & Thistlethwaite, P. J. Raman spectra and thermal transformations of ferrihydrite and schwertmannite. *J. Raman Spectrosc.* **33**, 104–111 (2002).

23. Gotić, M. & Musić, S. Mössbauer, FT-IR and FE SEM investigation of iron oxides precipitated from FeSO4 solutions. *J. Mol. Struct.* **834–836**, 445–453 (2007).

24. Xiao, Y. *et al.* Normal Mode Analysis of *Pyrococcus furiosus* Rubredoxin via Nuclear Resonance Vibrational Spectroscopy (NRVS) and Resonance Raman Spectroscopy. *J. Am. Chem. Soc.* **127**, 14596–14606 (2005).

25. Xiao, Y. *et al.* Dynamics of an [Fe4S4(SPh)4]2? cluster explored via IR, Raman, and nuclear resonance vibrational spectroscopy (NRVS)-analysis using 36S substitution, DFT calculations, and empirical force fields. *Dalton Trans.* 2192 (2006) doi:10.1039/b513331a.

26. Mitra, D. *et al.* Dynamics of the [4Fe-4S] Cluster in *Pyrococcus furiosus* D14C Ferredoxin via Nuclear Resonance Vibrational and Resonance Raman Spectroscopies, Force Field Simulations, and Density Functional Theory Calculations. *Biochemistry* **50**, 5220–5235 (2011).

27. Guo, Y. *et al.* Characterization of the Fe Site in Iron−Sulfur Cluster-Free Hydrogenase (Hmd) and of a Model Compound via Nuclear Resonance Vibrational Spectroscopy (NRVS). *Inorg. Chem.* **47**, 3969–3977 (2008).

28. Hassan, A., Macedo, L. J. A., Souza, J. C. P. D., Lima, F. C. D. A. & Crespilho, F. N. A combined Far-FTIR, FTIR Spectromicroscopy, and DFT Study of the Effect of DNA Binding on the [4Fe4S] Cluster Site in EndoIII. *Sci. Rep.* **10**, 1931 (2020).
